# Effects of Speed-Based Interventions to Reduce Bradykinesia in Individuals with Parkinson’s Disease: A Systematic Review Protocol

**DOI:** 10.3390/brainsci14121198

**Published:** 2024-11-28

**Authors:** Poliana Benfica, Aline Scianni, Jordana Magalhães, Sherindan Brito, Júlia Martins, Christina Faria

**Affiliations:** 1 Department of Physical Therapy, Universidade Federal de Minas Gerais (UFMG), Belo Horizonte 31270-901, Minas Gerais, Brazil; poliana.benfica@gmail.com (P.B.); ascianni@task.com.br (A.S.); jordanamagalhaes.jpm@gmail.com (J.M.); sherindanayessa@hotmail.com (S.B.); 2 Department of Physical Therapy, Faculdade Ciências Médicas de Minas Gerais (FCMMG), Belo Horizonte 30130-110, Minas Gerais, Brazil; julcmart@gmail.com

**Keywords:** Parkinson’s disease, bradykinesia, experimental designs, systematic review

## Abstract

Background/Objectives: Bradykinesia is considered one of the most disabling motor symptoms in individuals with Parkinson’s disease (PD). Speed-based interventions are promising for reducing bradykinesia in this population. This systematic review aims to describe speed-based interventions that have been employed for reducing bradykinesia in individuals with PD and verify their effects. Methods: This systematic review protocol was carried out in accordance with the Preferred Reporting Items for Systematic Review and Meta-Analysis Protocols (PRISMA-P). Electronic searches were performed in MEDLINE, EMBASE, PEDro, LILACS and SciELO databases and gray literature sources. Experimental studies that investigated the effects of speed-based interventions on bradykinesia in individuals with PD will be considered. Two independent reviewers will screen the studies, extract data and assess risk of bias using Revised Cochrane risk-of-bias tool for randomized trials (RoB 2) and ROBINS-I (“Risk Of Bias In Non-randomised Studies—of Interventions”). Grading of Recommendations Assessment, Development and Evaluation (GRADE) will be used to assed the quality of evidence. Conclusions: This systematic review will offer information regarding which types of speed-based interventions have been investigated for reducing bradykinesia in individuals with PD. This information may be important for clinical decision making and will help identify gaps in the literature that may be useful for the definition of future research objectives and the planning of new trials. Ethical permission is not required for this study. Systematic review registration: this systematic review was registered with the International Prospective Register of Systematic Reviews (PROSPERO; registration number: CRD42024543673).

## 1. Introduction

Neurological conditions including neurodegenerative disorders are considered the primary cause of disability and the second leading cause of mortality in the world [1,2]. Parkinson’s disease (PD) is among the most prevalent neurodegenerative disorders and its prevalence has increased considerably in recent decades [3]. Individuals with PD are characterized by presenting motor symptoms including rigidity, tremor, postural instability, and bradykinesia [4].

Among the motor symptoms, bradykinesia is considered one of the most disabling, affecting more than 80% of individuals with PD [4]. The term “bradykinesia” has been used to refer to the slow execution of voluntary movements [5]. However, bradykinesia also includes reduced range of movement (hypokinesia), the inability to initiate movement or the absence of movement (akinesia) [6], and amplitude and velocity decrement with repetitive and continuing movements (sequence effect) [7,8]. Bradykinesia affects the mobility, independence, and quality of life of individuals with PD [9,10,11] and, therefore, should be regarded in the treatment of this population.

Among the different types of treatments for bradykinesia, those involving exercises, specifically speed-based interventions are beneficial for individuals with PD and have the potential to reduce bradykinesia [12]. Speed-based training is considered a physical intervention that uses velocity feedback to prescribe and/or manipulate training load [13], i.e., that involves increasing the speed variable during its execution and progression. In addition, speed-based training improves other speed-related outcomes such as muscle power and mobility [14,15]. Since the end of the 1990s, studies about the effects of speed-based interventions in individuals with PD have been developed [16] in the modalities of aerobic exercise (treadmill [17] and bicycle training [12,18]), resistance exercises (muscle power training [14,15]), specific exercises (virtual reality–based training) [19] and robot-assisted gait training) [20]), and functional exercises (based on functional tasks [16]). Positive results of these interventions have been described for bradykinesia in the modalities of bicycle training [12,18] and muscle power training [14].

A systematic review in this area is necessary, considering the importance of bradykinesia in the health and functionality of individuals with PD and that speed-based interventions are promising for reducing bradykinesia in individuals with PD. As far as we are aware, no systematic reviews have been conducted about speed-based interventions in individuals with PD. Furthermore, despite recognizing the importance of investigating interventions that involve speed, guidelines for PD [21,22] and systematic reviews about physical exercise [23] and physiotherapy for PD [24] do not include speed-based interventions as a different category of exercise, likely because they emphasize other motor outcomes such as muscle strength, balance, and others. However, speed is an essential component for activities that require quick reactions. Neglecting this aspect can leave an important area of motor performance unexamined. Another pertinent piece of information is understanding what protocols are suitable for different speed-based interventions. Finally, it is necessary to carry out a critical analysis of the effects of interventions based on the methodological quality of the studies. Therefore, the current systematic review aims to describe speed-based interventions that have been employed for reducing bradykinesia in individuals with PD, to evaluate their effects and, therefore, help guide physiotherapists in clinical practice. Finally, gaps in the literature will be identified to facilitate the planning and design of new clinical trials.

## 2. Materials and Methods

This systematic review protocol was written following Preferred Reporting Items for Systematic Review and Meta-Analysis Protocols (PRISMA-P) [25,26] (Supplementary File S1) and the results will be reported following the Preferred Reporting Items for Systematic Review and Meta-Analysis (PRISMA 2020 statement) [27]. This systematic review protocol was previously registered in the International Prospective Register of Systematic Reviews (PROSPERO) (registration number CRD42024543673).

### 2.1. Eligibility Criteria

#### 2.1.1. Study Design

All experimental studies that investigated the effects of speed-based interventions aimed at reducing bradykinesia in individuals with PD will be considered for this study. Qualitative studies and systematic reviews will be excluded; however, their reference lists will be examined for relevant studies.

#### 2.1.2. Participants

Studies including participants aged 18 or older with a diagnosis of PD in any level of severity (1–5) according to the Hoehn and Yahr scale (HY) [28,29]. The authors of the studies involving mixed groups will be contacted to obtain specific data related to the individuals with PD. If specific data are unavailable, the study will be excluded.

#### 2.1.3. Interventions

Studies that included speed-based interventions aimed at reducing bradykinesia employing any type and mode of delivery will be included.

#### 2.1.4. Comparators

Studies with any type of control group, including no speed-based interventions, standard care, usual care, health education, counseling, self-management, behavioral interventions, waitlists, and no intervention are eligible. Control groups consisting of individuals without PD will be excluded. Based on the final articles included, the results will be grouped according to the type of control intervention.

#### 2.1.5. Outcomes

Studies that quantified bradykinesia by any subjective method (such as clinical rating scales, e.g., MDS Unified Parkinson’s Disease Rating Scale (MDS-UPDRS) part III [29], and Modified Bradykinesia Rating Scale (MBRS) [30]), objective method (technology-based tools, e.g., gait analysis systems and actigraphy [31]) and indirect method (through timed functional tests considered as surrogate measures of bradykinesia, e.g., -hole peg test, 4-square step test, timed-up and go (TUG) [12]) will be included.

### 2.2. Search Strategy

A comprehensive electronic search was conducted in the Medical Literature Analysis and Retrievel System Online (MEDLINE via PubMed), Excerpta Medica (EMBASE), Physiotherapy Evidence Database (PEDro), Literatura Latino-Americana e do Caribe em Ciências da Saúde (LILACS) and Scientific Electronic Library Online (SCIELO) databases, from their inception to April 2024, without any language limitations. The search strategy was developed based on previous studies with the guidance of an experienced researcher. The research strategy related to the population with PD was developed based on a systematic review developed by Elbers et al. 2015 [32]. The research strategy related to type of study (experimental studies) was based on two previously published reviews [32,33] and on mesh terms. The search strategy related to bradykinesia was based on mesh terms and definitions of the term bradykinesia [6]. The search strategy in each database is provided in Appendix A. Gray literature sources were also searched. Finally hand searches of the reference lists of included studies were conducted.

### 2.3. Screening of the Studies

Initially, database searches were carried out using the designed search strategy. The search results were saved and maintained in Rayyan Systems Inc. software (Cambridge, MA, USA) [34]. Two independent reviewers (PAYB, JPM) will screen all the retrieved titles and abstracts from the electronic search, according to the previously described inclusion criteria, and remove duplicate studies. Full texts will be screened by the same reviewers (PAYB, JPM), independently. Any disagreement will be settled by discussion and consensus. A third reviewer (CDCMF) will be consulted, if needed. All the reasons for exclusion of ineligible studies will be documented. The results of the screening process will be presented in details using the PRISMA information flow [27].

### 2.4. Data Extraction

Two independent reviewers (PAYB, JPM) extracted data from all included studies. The relevant data extracted from all the included studies will be compiled and presented in tables. Disagreements between reviewers will be resolved by discussion and consensus. Data extraction will include: (1) study details: authors, year of publication, setting, and type of study (randomized clinical trial (RCT) or other type of experimental study); (2) sample characteristics: number of participants, age, sex, time since the onset of the PD, the stage of the disease using the H&Y; (3) methods: study design, sampling, and comparison/control group; (4) interventions: intervention details, duration, frequency, intensity, length and supervision; (5) primary outcome (bradykinesia) and other outcomes: description, measurement instruments, intervention effects on the outcomes; (6) other information: safety and adverse effects. If needed, any additional information expressing a conflict of interest or bias will be also extracted. The corresponding author of the studies with missing or incomplete data will be contacted for clarification. Disagreements will be resolved through discussion with the third reviewer (CDCMF).

### 2.5. Risk of Bias

The methodological quality regarding the risk of bias of the studies will assessed by Revised Cochrane risk-of-bias tool for randomized trials (RoB 2) for RCTs and by ROBINS-I (“Risk Of Bias In Non-randomised Studies—of Interventions”) for non-randomized studies [35,36]. RoB 2 has five different domains used to generate the overall risk of bias from RCTs. The risk of bias judgments for each domain are “low risk of bias”, “some concerns”, or “high risk of bias” [35]. The overall risk of bias will be classified as: “Low risk of bias” (low risk of bias for all domains), “Some concerns” (some concerns in at least one domain), “High risk of bias” (high risk of bias in at least one domain or some concerns for multiple domains) [35].

ROBINS-I presents seven domains that provide the risk of bias from studies that did not use randomization to assign interventions [36]. The classifications for risk of bias judgments are “Low risk” (low risk of bias for all domains), “Moderate risk” (low or moderate risk of bias for all domains), “Serious risk” (serious risk of bias in at least one domain), and “Critical risk” (critical risk of bias in at least one domain) [36]. The methodological quality regarding the risk of bias of the studies will be evaluated case-by-case independently by two reviewers (PAYB, JPM). Disagreements will be resolved by discussion and consensus. If needed, a third reviewer (CDCMF) will be consulted. Study authors will be contacted if the reports lack sufficient details to assess the risk of bias. During qualitative synthesis, studies classified as “high risk of bias” (RoB 2) or “serious/critical risk” (ROBINS-I) will be discussed separately or excluded from the primary analyses, depending on the context and heterogeneity of the results.

### 2.6. Quality of Evidence

The quality of the evidence of the studies will be assessed using the Grading of Recommendations Assessment, Development and Evaluation (GRADE) [37]. GRADE classifies the quality of the studies as high (further research is very unlikely to change the confidence in the effect estimates), moderate (further research is likely to have an important impact on the confidence in the effect and may change the estimate), low (further research is very likely to have an important impact on the confidence in the effect and is likely to alter the estimate), and very low (any estimate of the effect is very uncertain) [37].

### 2.7. Strategy for Data Synthesis

This systematic review also includes a qualitative synthesis, presenting information, in text and tables, to summarize the results of the included studies. A narrative synthesis will be conducted to examine the results and explore associations within and between the included trials. If the studies were sufficiently homogeneous in terms of interventions and outcomes, and if adequate data are available, forest-plots and meta-analyses will be performed. These analyses aim to synthesize the direction, magnitude, and consistency of potential effects using Review Manager software (RevMan) (Review Manager (RevMan) [Computer program]. Version 5.3. Copenhagen, Denmark: The Nordic Cochrane Centre, The Cochrane Collaboration, 2014).

### 2.8. Analyses of Subgroups or Subsets

All analyses will be conducted considering two primary groups of studies: RCTs and other experimental studies. The subgroup analyses will be also carried out, if sufficient data was available. These analyses examined differences between bradykinesia measurements (e.g., subjective vs. objective instruments), type, duration and delivery of the speed-based intervention, group comparisons, quality, and risk of bias.

### 2.9. Investigation of Heterogeneity

Meta-analyses will be performed if there were at least two homogeneous RCTs (studies that investigated the effect of the same intervention and reported the results in a similar way). The I2 statistic will be used to measure heterogeneity between studies. The cutoff point used for significant heterogeneity will be >50% [38].

### 2.10. Sensitivity Analysis

To examine the robustness of meta-analyses, sensitive analyses will be performed. If heterogeneity was high (I^2^ > 50%), sensitivity analyses will be conducted for primary outcomes following the parameters: (1) exclusion of studies identified as having a high risk of bias; and (2) exclusion of studies with missing data, when these could not be provided by the study authors.

## 3. Discussion

As far as we are aware, this is the first systematic review that aimed to describe speed-based interventions employed to reduce bradykinesia in individuals with PD and investigate their effects. The negative impact of bradykinesia on the health and functionality of individuals with PD is well-recognized [4,7]. Therefore, treatment aimed at reducing bradykinesia is mandatory for those individuals. Bradykinesia is closely linked to the speed of movement because it relates to a decrease in movement speed or slowness of movement [7,8]. It is therefore essential to consider the speed of movement in any exercise modality to reduce bradykinesia in individuals with PD [39,40].

This systematic review will provide a rigorous description of speed-based interventions that have been investigated for reducing bradykinesia in individuals with PD. In addition, the effects of this intervention will be pointed out. In this context the GRADE method is a useful tool for assessing the quality of evidence, considering factors such as risk of bias, imprecision, consistency, and applicability of the included studies [37]. When there is significant heterogeneity between subgroups, it can directly impact the confidence in effect estimates because inconsistent results between subgroups may indicate that un-controlled factors are influencing the observed effects. If sufficiently homogeneous data are available to conduct meta-analyses, clinicians will gain insights into the expected effect size associated with specific intervention. Therefore, the results found can help clinicians in clinical decision-making as well as in planning their interventions. Moreover, these results can help translate evidence into practical and scalable strategies for clinical application, including adapting interventions for resource-limited settings and optimizing their integration into existing rehabilitation programs. Furthermore, this systematic review may help identify gaps in the literature, particularly regarding the types and specific intervention protocols, group comparisons, measurement instruments, and both short-term and long-term effects. Therefore, the results found will be valuable for the definition of future research objectives and informing the planning of new research trials.

Finally, the findings from this systematic review will be disclosed through peer-reviewed scientific publications and conference presentations at scientific events. If the protocol needs to be amended following its publication, amendments will be recorded in the PROSPERO registry, and any deviations from the protocol will be detailed in the final published review paper.

## Data Availability

Not applicable.

## Supplementary Materials

The following supporting information can be downloaded at: https://www.mdpi.com/article/10.3390/brainsci14121198/s1, Additional Supplementary Materials File S1: PRISMA-P (Preferred Reporting Items for Systematic review and Meta-Analysis Protocols) 2015 checklist: recommended items to address in a systematic review protocol).

## Appendix A

**Table A1:** Search Strategy for Each Database.

Database	Search Strategy
MEDLINE	(((((((((((((((exp parkinson disease/) OR (exp parkinsonism/)) OR (parkinsonian disorders.ti,ab.)) OR (parkinsonism.ti,ab.)) OR (parkinson.ti,ab.)) OR (parkinson disease.ti,ab.)) OR (parkinsons disease.ti,ab.)) OR (parkinson’s disease.ti,ab.)) OR (idiopathic parkinson disease.ti,ab.)) OR (idiopathic parkinsonsdisease.ti,ab.)) OR (idiopathic parkinson’s disease.ti,ab.)) OR (“parkinson disease”)) OR (Parkinsonism)) OR (parkinson*)) AND ((((((((((((((((((((((((((((((((((((((((((((randomized controlled trial.pt.) OR (randomized controlled trials.mp. or Randomized Controlled Trial/)) OR (random allocation.mp. or Random Allocation/)) OR (randomization/)) OR (random*.mp.)) OR (controlled study/)) OR (clinical trial/)) OR (controlled clinical trial.pt.)) OR (controlled clinical trials.mp. or controlled clinical trial/)) OR (double blind procedure/)) OR (double-blind method.mp. or Double-Blind Method/)) OR (single blind procedure/)) OR (single-blind method.mp. or Single-Blind Method/)) OR (crossover procedure/)) OR ((clinica* adj3 trial*).mp)) OR ((((singl* or doubl* or trebl* or tripl*) adj3 (mask* or blind* or method*)).mp.))) OR (exp placebo/)) OR (placebos.mp. or Placebos/)) OR (placebo*.mp. or Placebos/)) OR ((((control* or prospectiv*) adj3 (trial* or method* or stud*)).mp.))) OR (((crossover* or cross-over*).mp.))) OR (research design.mp. or Research Design/)) OR (multicenter study.pt.)) OR (intervention studies.mp. or Intervention Studies/)) OR (cross-over studies.mp. or Cross-Over Studies/)) OR (control*.tw.)) OR (exp evaluation studies/)) OR (follow-up studies.mp. or Follow-Up Studies/)) OR (versus.tw.)) OR (“experimental studies”)) OR (“experimental designs”)) OR (“quasi-experimental studies”)) OR (“quasi experimental studies”)) OR (“quasi-experimental study”)) OR (“nonrandomized clinical trial”)) OR (“nonrandomized clinical trials”)) OR (“non-randomized clinical trial”)) OR (“non-randomized clinical trials”)) OR (“nonrandomized controlled trial”)) OR (“nonrandomized controlled trials”)) OR (“non-randomized controlled trial”)) OR (“non-randomized controlled trials”)) OR (“non randomized controlled trial”)) OR (“non randomized controlled trials”))) AND ((((((((bradykinesia) OR (hypokinesia)) OR (akynesia)) OR (bradykine*)) OR (hypokine*)) OR (akine*)) OR (“sequence effect”)) OR (slowness))
EMBASE	(‘parkinson disease’/exp OR ‘parkinson disease’ OR ‘parkinsonism’/exp OR ‘parkinsonism’ OR ‘parkinsonian disorders’ OR ‘idiopathic parkinson disease’/exp OR ‘idiopathic parkinson disease’ OR ‘idiopathic parkinsonsdisease’) AND (‘randomized controlled trial’/exp OR ‘randomized controlled trial’ OR ‘randomized controlled trials’ OR ‘randomization’/exp OR ‘randomization’ OR ‘random allocation’ OR ‘controlled study’/exp OR ‘controlled study’ OR ‘clinical trial’/exp OR ‘clinical trial’ OR ‘controlled clinical trial’/exp OR ‘controlled clinical trial’ OR ‘double blind procedure’/exp OR ‘double blind procedure’ OR ‘double-blind method’ OR ‘single blind procedure’/exp OR ‘single blind procedure’ OR ‘single-blind method’ OR ‘crossover procedure’/exp OR ‘cross-over studies’ OR ‘placebo’/exp OR ‘placebo’ OR ‘placebos’ OR ‘multicenter study’/exp OR ‘multicenter study’ OR ‘intervention studies’/exp OR ‘intervention studies’ OR ‘evaluation studies’/exp OR ‘evaluation studies’ OR ‘follow-up’/exp OR ‘follow-up’ OR ‘experimental study’/exp OR ‘experimental study’ OR ‘experimental studies’ OR ‘experimental design’/exp OR ‘experimental design’ OR ‘quasi experimental study’/exp OR ‘quasi experimental study’ OR ‘quasi-experimental studies’ OR ‘nonrandomized clinical trial’ OR ‘nonrandomized clinical trials’ OR ‘non-randomized clinical trial’ OR ‘non-randomized clinical trials’ OR ‘nonrandomized controlled trial’ OR ‘nonrandomized controlled trials’ OR ‘non-randomized controlled trial’ OR ‘non-randomized controlled trials’ OR ‘non randomized controlled trial’ OR ‘non randomized controlled trials’) AND (‘bradykinesia’/exp OR ‘bradykinesia’ OR ‘hypokinesia’/exp OR ‘hypokinesia’ OR ‘akynesia’ OR ‘sequence effect’ OR ‘slowness’)
PEDro	1. parkinson* AND bradykine*; 2. parkinson* AND hypokine*; 3. parkinson* AND akyne* 4. parkinson* AND ‘sequence effect’; 5. parkinson* AND slowness; 6. ‘parkinson disease’ AND bradykine*; 7. ‘parkinson disease’ AND hypokine*; 8. ‘parkinson disease’ AND akyne*; 9. ‘parkinson disease’ AND ‘sequence effect’; 10. ‘parkinson disease’ AND slowness; 11. ‘parkinson disorders’ AND bradykine*; 12. ‘parkinson disorders’ AND hypokine*; 13. ‘parkinson disorders’ AND akyne*; 14. ‘parkinson disorders’ AND ‘sequence effect’; 15. ‘parkinson disorders’ AND slowness
LILACS	((“parkinsonian disorders”) OR (“parkinson disease”) OR (“parkinson disorders”) OR (parkinsonism) OR (parkinson) OR (“parkinsons disease”) OR (“parkinson’s disease”) OR (“idiopathic parkinson disease”) OR (“idiopathic parkinsons disease”) OR (“idiopathic parkinson’s disease”)) AND ((“randomized controlled trial”) OR (“randomized controlled trials”) OR (“random allocation”) OR (randomization) OR (random*) OR (“controlled study”) OR (“clinical trial”) OR (“controlled clinical trial”) OR (“controlled clinical trials”) OR (“double blind procedure”) OR (“double-blind method”) OR (“single blind procedure”) OR (“single-blind method”) OR (“crossover procedure”) OR (clinica*) OR (trial*) OR (singl*) OR (doubl*) OR (trebl*) OR (tripl*) OR (mask*) OR (blind*) OR (placebo) OR (placebo*) OR (control*) OR (crossover*) OR (cross-over*) OR (“multicenter study”) OR (“intervention studies”) OR (“cross-over studies”) OR (“evaluation studies”) OR (“follow-up studies”) OR (“experimental studies”) OR (“experimental designs”) OR (“quasi-experimental studies”) OR (“quasi experimental studies”) OR (“quasi-experimental study”) OR (“nonrandomized clinical trial”) OR (“nonrandomized clinical trials”) OR (“non-randomized clinical trial”) OR (“non-randomized clinical trials”) OR (“nonrandomized controlled trial”) OR (“nonrandomized controlled trials”) OR (“non-randomized controlled trial”) OR (“non-randomized controlled trial”) OR (“non randomized controlled trial”) OR (“non randomized controlled trials”)) AND ((bradykinesia) OR (hypokinesia) OR (akynesia) OR (bradykine*) OR (hypokine*) OR (akine*) OR (“sequence effect”) OR (slowness))
SciELO	1. (parkinson*) AND (bradykine*); 2. (parkinson*) AND (hypokine*); 3. (parkinson*) AND akyne*); 4. (parkinson*) AND (sequence effect); 5. (parkinson*) AND (slowness); 6. (parkinson disease) AND (bradykine*); 7. (parkinson disease) AND (hypokine*); 8. (parkinson disease) AND (akyne*); 9. (parkinson disease) AND (sequence effect); 10. (parkinson disease) AND (slowness); 11. (parkinson disorders) AND (bradykine*); 12. (parkinson disorders) AND (hypokine*); 13. (parkinson disorders) AND (akyne*); 14. (parkinson disorders) AND (sequence effect); 15. (parkinson disorders) AND (slowness)
